# Prognostic value of CEA/CA72-4 immunohistochemistry in combination with cytology for detecting tumor cells in peritoneal lavage in gastric cancer

**DOI:** 10.7150/jca.47113

**Published:** 2020-09-01

**Authors:** Linlin Qu, Liang He, Zhifang Jia, Quan Wang

**Affiliations:** 1 Department of Laboratory Medicine, the First Hospital of Jilin University, Jilin, China;; 2 Department of Gastrointestinal Surgery, the First Hospital of Jilin University, Jilin, China;; 3 Department of Clinical Research, the First Hospital of Jilin University, Jilin, China.

**Keywords:** Gastric Cancer, Peritoneal Lavage, Metastasis, Prognosis, Immunohistochemistry.

## Abstract

**Aim:** To study the value and efficiency of CEA/CA72-4 immunohistochemistry in detecting free tumor cells from peritoneal lavage, in order to provide reliable lab information for subsequent intraperitoneal chemotherapy. **Methods:** A total of 112 progressive gastric cancer patients were enrolled from Oct. 2016 to Oct 2017, who were pathologically diagnosed as gastric cancer after surgery. Peritoneal lavage was respectively collected during operation. Cytology and CEA/CA72-4 immunohistochemistry of peritoneal lavage samples was performed. Overall survival and recurrence free survival was analyzed. **Results:** Cytology showed 16 positive cases (14.29%), CEA immunohistochemistry showed 29 positive cases (25.89%), CA72-4 immunohistochemistry showed 33 positive cases (29.46%). McNemar's test showed significant difference in positivity between cytology (CY+) and CEA/CA72-4 immunohistochemistry (IHC+). Kappa test showed consistency between immunohistochemistry of CEA and CA72-4 with cytology. Patients with CY+/IHC+ had the poorest overall survival (OS) as well as recurrence free survival (RFS), followed by those with CY+ or IHC+, while those with CY-/IHC- had higher OS and RFS. The differences of OS and RFS in IHC+ group were worse than that in IHC- group. Kaplan-Meier analysis showed that positive CEA/CA72-4 IHC revealed poorer prognosis than the negative cases. **Conclusions:** Due to the limitation of cytology, combination of cytology and immunohistochemistry appears to be more efficient for predicting prognosis of progressive gastric cancer.

## INTRODUCTION

In recent years, mortality of gastric cancer has reduced due to radical gastrectomy accompanied by adjuvant chemotherapy. However, in worldwide, gastric cancer is still the third leading cause of cancer death 1. Clinical decisions of post-adjuvant treatment for gastric cancers are mainly depended on tumor invasive depth, TNM staging, and whether lymph nodes metastasis and/or peritoneal metastasis or not 2. Thus, besides traditional pathology, the detection of free tumor cells in peritoneal lavage appears to be very important in post-adjuvant decision making for those advanced gastric cancers.

The aim of this study is to confirm the presence of the small amount of free tumor cells in peritoneal lavage, to evaluate methods of detection for these cells, and to explore the value of detecting free tumor cells in prognosis and post-adjuvant decision making.

## MATERIALS AND METHODS

### Patients and Clinical Treatments

In this study, 112 patients with advanced GC (GC: gastric cancer) who underwent open or laparoscopic gastrectomy as first treatment in the First Hospital of Jilin University from Oct. 2016 - Oct. 2017 were included in the study, who were pathologically diagnosed and other tumors were excluded. All the patients had radical gastrectomy of gastric cancer with pathology-proven negative cutting edges.

According to the seventh AJCC (AJCC: American Joint Committee on Cancer)/International Union Against Cancer, TNM system 3 was used for the staging of gastric cancer.

In GC group, patients with positive cytological findings received intraperitoneal chemotherapy after surgery. Paclitaxel was injected intravenously (50 mg/m^2^) on day 1 and intraperitoneally (diluted in 1.0 L of normal saline, 20 mg/m^2^) on day 8. Take S-1 (80 mg/m^2^) orally, daily (1-14 days, rest for 1 week). Treatment was repeated every 3 weeks until the disease progress or intolerable toxicity occurred.

### Ethical Declaration

This study was performed according to the Declaration of Helsinki laid down in 1964 and was approved by the Ethics Committee of the First Hospital of Jilin University. Patients were informed before operation and voluntarily participated in the study. Privacy of patients was conserved.

### Peritoneal Lavage Collection

At the first time when laparoscopic puncture was set into the peritoneal cavity, wash gastric bed, spleen fossa and Douglas pouch with 500ml of warm saline, and then aspirated out to a clean container, which was designated as the first PLs (PLs: peritoneal lavage samples). Drain directly for those who already had ascites. After the complete removal of the tumor or lesion, the peritoneal cavity was washed and aspirated. PL samples were centrifuged under 1500rpm, 5min at room temperature to collect intact cells. The precipitates were used for cytology and immunohistochemistry.

### Pathology and Cytology Examination

**Figure 1:**
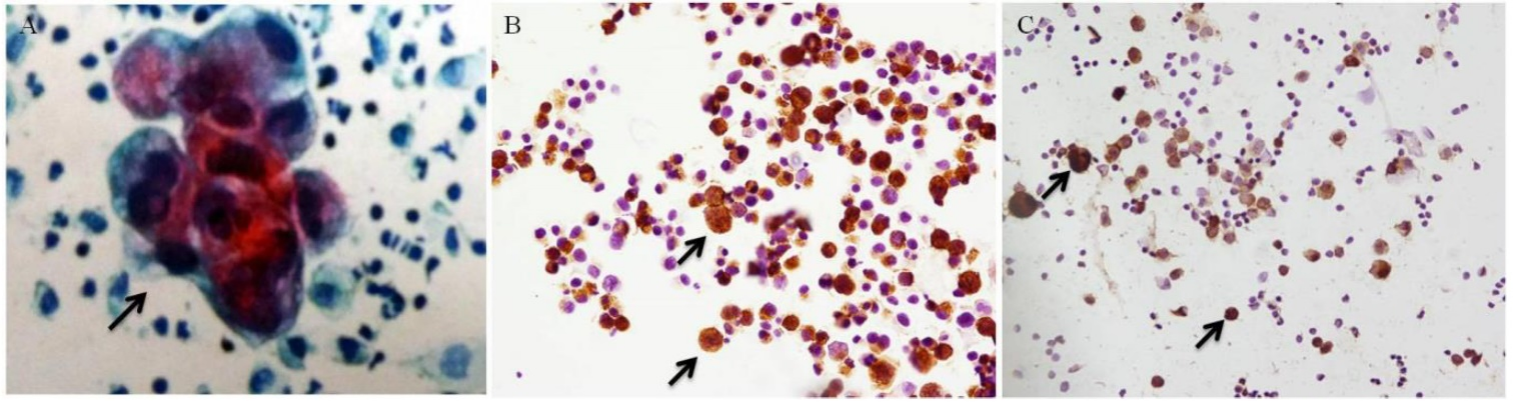
Positive control of cytology and immunohistochemistry (black arrows, ×200). (A) Positive cytology under Pap staining. (B) Positive CA72-4 immunohistochemistry. (C) Positive CEA immunohistochemistry.

After surgery, the tumors underwent pathological examination. HE staining was used on the slides from the paraffin blocks of each sample. The precipitates of PLs were smeared onto several slides by Autoslide Slide Maker Stainer (Simens, SN ABX15091316). Cytology was done followed by Pap staining using hematoxylin (Sigma, CAS 517-28-2). A positive result for cytology was marked as CY+ (Figure 1 A), while CY- for a negative cytology result.

### Immunohistochemistry Staining and Evaluation

IHC (IHC: Immunohistochemistry) was used to examine the expression of CEA and CA72-4 in tumor cells from PLs. A cytologically positive slides was taken as positive control (Figure 1 B/C), and a non-cancer PL sample was taken as negative control. Slides of PLs were fixed with 4% paraformaldehyde, then put in 3% H_2_O_2_ to exhaust endogenous peroxidase; slides were incubated overnight in 4°C with primary antibodies, rabbit monoclonal anti-CEA (Abcam, Cambridge, MA) and rabbit monoclonal anti-CA72-4 (Abcam, Cambridge, MA), and then goat anti-rabbit IgG secondary antibodies (Abcam, Cambridge, MA) for 1 hr. After incubation with the appropriate HRP (HRP: horseradish peroxidase) - conjugated antibodies (Bioworld Technology, St. Louis Park, MN) for 2 hours, the signals were observed with a Diaminobenzidine (DAB) kit, stained with hematoxylin. The IHC slides were observed through microscope.

Cytoplamic CEA and CA72-4 expression inside free cancer cells of PLs were evaluated and scored using microscopy. By assessing the proportion of positive cells among the whole cells, scores were recorded as A: 0 (<5%), 1 (6-25%), 2 (26-50%), 3 (51-75%), 4 (76-100%); the intensity of staining were graded as B: 0 (negative), 1 (weak staining), 2 (medium staining), 3 (strong staining). The final score for each sample was calculated by average A × B of successive 10 high power fields. A final score >1 was considered as positive and marked as IHC+, otherwise marked as IHC-.

### Follow-up

GC patients were followed up 2.4-24 months after surgery until death or the scheduled deadline. Examination for patients included CT scan and gastroscopy. CT scan and gastroscopy was performed every six months. All CT images were validated by an experienced radiologist who was informed of the patients' pathological diagnosis but blinded to the disease progression. Symptoms of peritoneal metastasis within 6 months after surgery were all regarded as peritoneal metastasis before surgery. Patients with positive cytology during surgery or peritoneal metastasis within 6 months after surgery were all classified as the peritoneal metastasis group, which was used as the golden standard to measure the value of immunohistochemistry. Rates of true positive, true negative, false positive and false negative were recorded. Gastroscopy results were provided by an experienced physicist. CR (CR: complete response), PR (PR: partial response), SD (SD: stable disease), and PD (SD: stable disease) were assessed according to the RECIST Criteria: version 1.1 4.

### Statistical Analysis

By SPSS 23.0 software, the positive rates of immunohistochemistry and cytology were compared using McNemar's test. Kappa coefficient was used to measure the consistency of results from CEA/CA72-4 IHC and cytology. The sensitivity and specificity to identifying peritoneal metastasis were calculated. The survival curves were plot using Kaplan-Meier method for both OS (OS: overall survival) and RFS (RFS: recurrence free survival), and then compared using log-rank test. Statistical significance was considered as two-sided* P* value <0.05.

## RESULTS

### Patients' Information

The clinical and pathological information of the patients was summarized in Table 1.

### Positivity and Consistency of CEA/CA72-4 Cytology and Immunohistochemistry

The positive control of cytology and CEA/CA72-4 immunohistochemistry showed in Figure 1. Among the 112 cases, CEA immunohistochemistry showed 29 positive cases (25.89%) and 83 negative cases (74.11%), while CA72-4 immunohistochemistry showed 33 positive cases (29.46%) and 89 negative cases (79.46%) (Table 2). McNemar's test showed no significant difference between CEA and CA72-4 immunohistochemistry (*p*=0.219). Cytology showed 16 positive cases (14.29%) and 96 negative cases (85.71%), among which there were 18 immunochemistry (+) cases showing cytology (-). The total positive rate of immunohistochemistry was 30.36% (34/112) (Table 3). Positivity of immunohistochemistry was higher than cytology, and McNemar's test showed significant difference in positivity between cytology and immunohistochemistry (*p*<0.001). Kappa test showed consistency between immunohistochemistry with cytology was 0.866 (p<0.001).

### Sensitivity and Specificity of Cytology and Immunohistochemistry

In this study, peritoneal metastasis defined as positive cytology or peritoneal metastasis in 6 months after surgery. Until the end of follow up, there were 59 cases with peritoneal metastasis. True positive, true negative, false positive, false negative of cytology and immunohistochemistry were list in Table 4. Sensitivity, specificity and Youden index were calculated (Table 4).

### Survival Analysis Cytology and Immunohistochemistry

**Figure 2:**
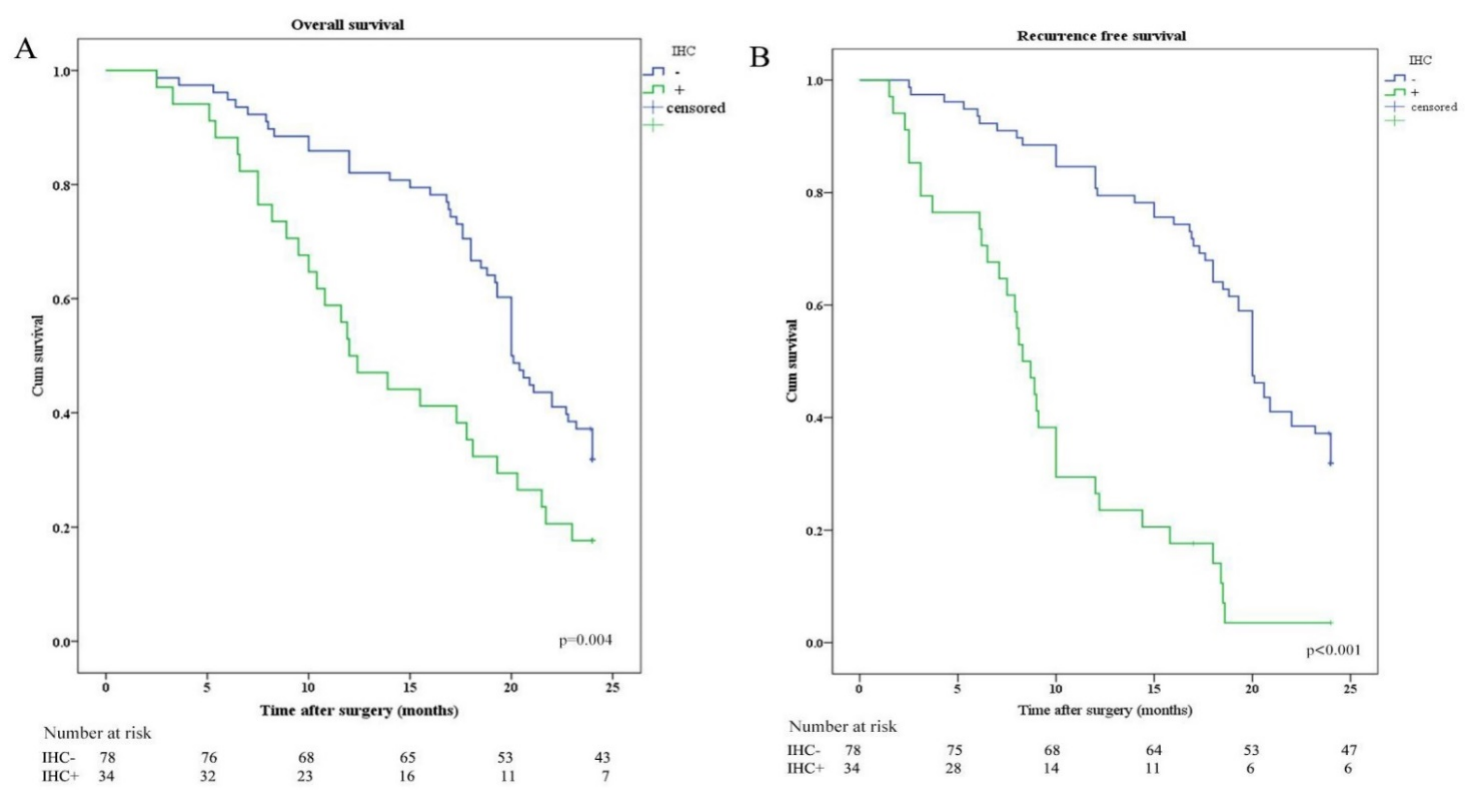
Kaplan-Meier analysis of OS and RFS stratified by IHC results. (A) Kaplan-Meier analysis of OS between IHC positive (IHC+) and IHC negative (IHC-). (B) Kaplan-Meier analysis of RFS between IHC positive (IHC+) and IHC negative (IHC-).

**Figure 3:**
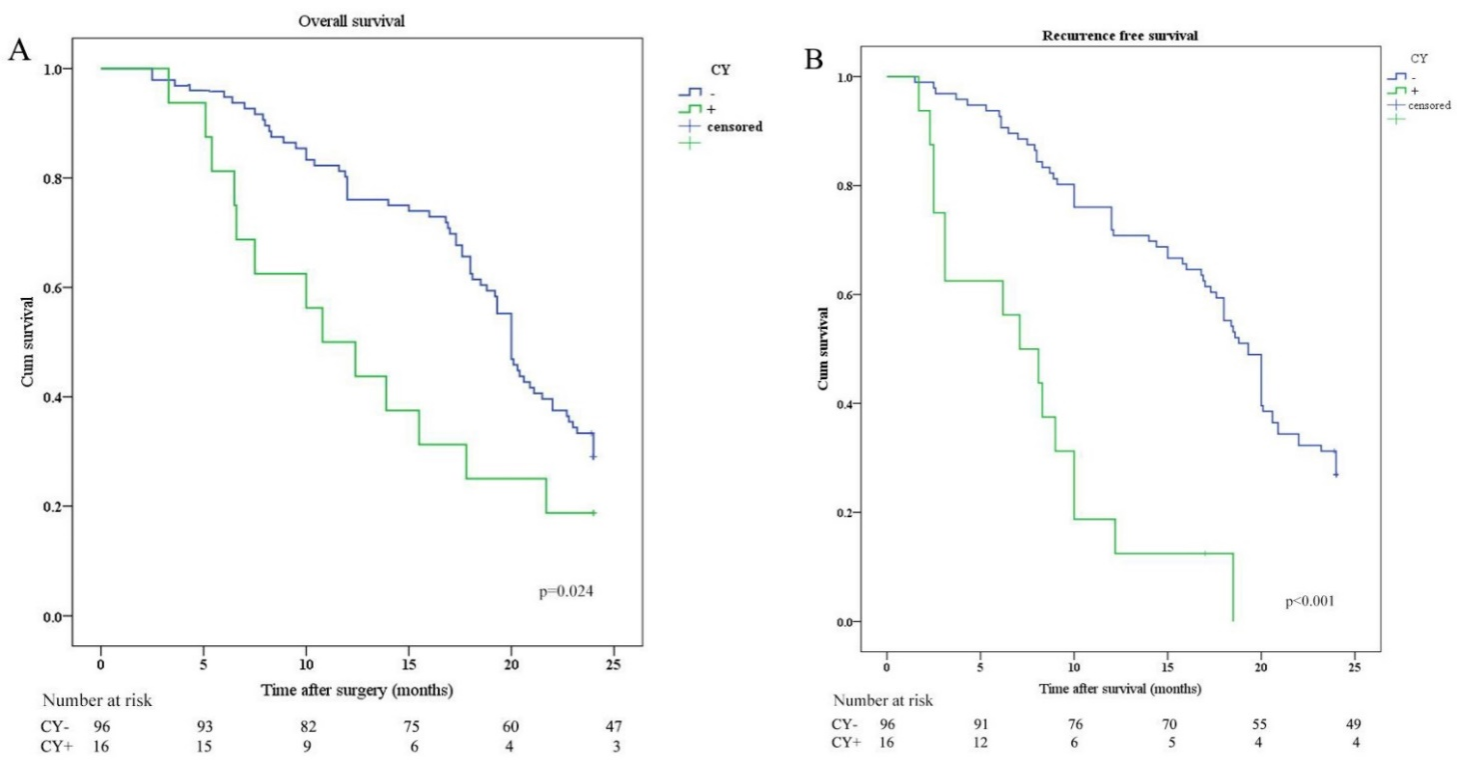
Kaplan-Meier analysis of OS and RFS stratified by CY results. (A) Kaplan-Meier analysis of OS between CY positive (CY+) and CY negative (CY-). (B) Kaplan-Meier analysis of RFS between CY positive (CY+) and CY negative (CY-).

**Figure 4:**
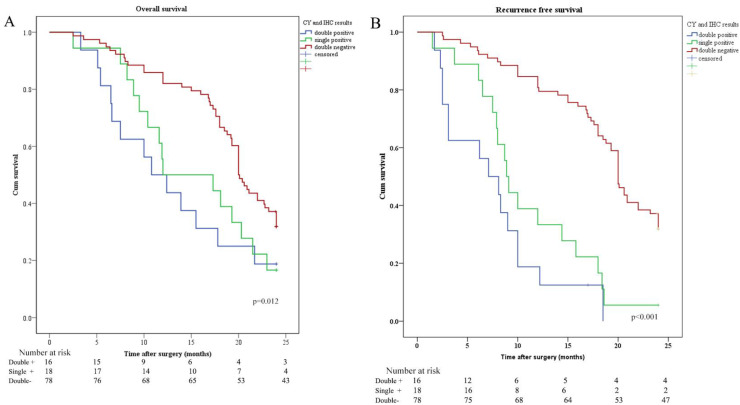
Kaplan-Meier analysis of OS and RFS stratified by CY and IHC results. (A) Kaplan-Meier analysis of OS between CY and IHC double positive (CY+/IHC+), single positive (CY+ or IHC+), and double negative (CY-/IHC-). (B) Kaplan-Meier analysis of RFS among CY+/IHC+, CY+ or IHC+, and CY-/IHC-.

OS and RFS were plotted according to CY (CY: cytology) and IHC of peritoneal lavage. Median OS in patients with CY-/IHC-, CY+ or IHC+, and CY+/IHC+ were 20.0 months, 12.0 months, and 10.8 months, respectively. In order to investigate the relationship between IHC and survival, the differences in OS and RFS between IHC+ and IHC- patients were analyzed and revealed that OS and RFS were worse in IHC+ group (Figure 2). The relationship between CY and survival was also analyzed, revealing that OS and RFS were worse in CY+ group (Figure 3). Patients with CY+/IHC+ ("double positive") had the poorest OS as well as RFS, followed by those with CY+ or IHC+("single positive"), while those with CY-/IHC-("double negative"), had higher OS and RFS, with statistically significant differences (Figure 4).

## DISCUSSION

GC is the fifth most common malignant cancer and the third leading cause of cancer death in the world 5. During 2008-2014, 5-year relative survival in the US is 31.0% for all stages, 68.1% for localized tumors, 30.6% for regional tumors, and 5.2% for distant tumors 6, which suggests that distant metastasis is the main threat to deaths in gastric cancer. Among distant metastasis, peritoneal metastasis is the most common in gastric cancer 7. The updated TNM Staging by AJCC (AJCC: American Joint Committee on Cancer) had classified peritoneal metastasis as M1c 8, 9, positive peritoneal lavage cytology is defined as M1 for staging by the Stomach Cancer Group of the Japan Clinical Oncology Group (JCOG) 10. Although cytological examination of peritoneal lavage is considered as a traditional method for detecting free tumor cells in peritoneal lavage 11, it is reported that detection rates were 2.2%~47.2% and more than half of positive cases were missed due to the limitations in the aspect of sensitivity. In recent years, some independent studies confirmed that intraperitoneal treatment strategy is necessary for the peritoneal metastatic cases 12, 13. The sensitivity of recognition for peritoneal metastasis can significantly improve patients' prognosis 14, 15. The need for precise diagnosis of metastasis lies in IP (IP: intraperitoneal chemotherapy) decision making, for example, a meta-analysis was carried out which showed IP can benefit patients' prognosis 16. Patients with gastric cancer under IP had a higher rate of negative lavage cytology 17. Thus, finding novel and useful biomarkers to improve the efficiency for detecting peritoneal metastasis is very important 18. Beside peritoneal cytology, genetic detection of CEA 19, CK20 (CK20: cytokeratin 20) 20, CK19 (CK19: cytokeratin 19) 21, and MAGE (MAGE: melanoma-associated gene) 22 using RT-PCR (RT-PCR: reverse transcriptase-polymerase chain reaction) were reported as one of available tools that correlated with patients' metastasis. Moreover, immunohistochemistry of cells from peritoneal lavage is considered another available strategy for directly detecting metastatic tumor cells.

In this study, we chose CEA and CA72-4 as candidate markers for immunochemistry examination to detect free tumor cells from peritoneal lavage for patients with gastric cancer. CEA expression in peritoneal lavage fluid was reported to have significantly poorer OS and RFS compared with those negative cases 7. Several evidence indicated that CEA was the most sensitive biomarker for analyzing gastric cancer 8, 9. However, the specificity of CEA is insufficient because it is widely synthesized and secreted by many tumors such as ovarian cancer, pancreatic carcinoma, colorectal cancer, and lung cancer. Yet there were no convincing partner markers as compensate for the weakness of CEA. Therefore, we tried to evaluate the efficiency of CA72-4 in the establishment of immunohistochemistry examination.

From the above findings, we conclude that CEA/CA72-4 immunohistochemistry may become a new method to evaluate peritoneal metastasis as an improvement for the limitations of cytology. The contribution of the study is to establish an alternative tool to predict peritoneal metastasis for patients with gastric cancer. Due to the feasibility of peritoneal lavage during radical gastrectomy, the application of CEA/CA72-4 immunohistochemistry emerges as a more sensitive indicator to predict the prognosis of these patients, as well as the timely decision making of post-adjuvant therapy. Objectively, this study has some limitations regarding on the relatively small sample size, heterogeneity of postoperative treatment. Further, a large-scale observation will be required to determine whether this examination can be widely adopted in the clinic.

## Figures and Tables

**Table 1:** Basic information and pathological features of GC patients (N=112).

Characteristics	Classification	Number of cases (%)
Age	≥60	60 (53.57)
	<60	52 (46.43)
Gender	Male	93 (83.04)
	Female	19 (16.96)
Tumor location	Fundus ventriculi	24 (21.43)
	Gastric angle/ Gastric antrum	88 (78.57)
Tumor size	≥5cm	59 (52.68)
	<5cm	53 (47.32)
Histology	Low differentiated	49 (43.75)
	Low-moderate differentiated	46 (41.07)
	Moderate differentiated	12 (10.71)
	Signet ring cell carcinoma	5 (4.47)
Lauren classification	Intestinal-type	21 (18.75)
	Mixed-type	40 (35.71)
	Diffused-type	51 (45.54)
Vascular/ neural invasion	Yes	106 (94.64)
	No	6 (5.36)
Depth of invasion	T1/T2	6 (5.36)
	T3	103 (90.18)
	T4	3 (2.68)
Lymph node metastasis	Yes	104 (92.86)
	No	8 (7.14)

**Table 2:** Results of CEA and CA72-4 immunohistochemistry examination (N=112).

CA72-4 Immunohistochemistry	CEA Immunohistochemistry		Total (n/%)
	positive	negative	
positive	28	5	33/29.46%
negative	1	78	79/70.54%
Total (n/%)	29/25.89%	83/74.11%	112/100%

**Table 3:** Results of immunohistochemistry and cytology (N=112).

Immunohistochemistry	Cytology		Total (n/%)
	positive	negative	
positive	16	18	34/30.36%
negative	0	78	78/69.64%
Total (n/%)	16/14.29%	96/85.71%	112/100%

**Table 4:** Results of cytology and immunohistochemistry in peritoneal metastasis (N=112).

Methods/ Results	Peritoneal metastasis		Sensitivity (%)	Specificity (%)	Youden index
	Positive	Negative			
Cytology					
Positive	16	0	59.26%	100.00%	0.6
Negative	11	85			
Immunohistochemistry					
Positive	27	7	100.00%	91.76%	0.9
Negative	0	78			
Total (n)	27	85			
